# Supplementary material for: Identification of Freezing-Responsive microRNAs and Their Targets in Chinese Jujube by Small RNA and Degradome Sequencing
Source: Int J Mol Sci. 2026 Sep 4;27(17):7912. doi: 10.3390/ijms27177912 (PMC13566890; doi:10.3390/ijms27177912)
Supplement: Supplementary file 1 [file ijms-27-07912-s001.zip › Supplementary Material S2/novel_miR_82.pdf]

Provisional ID : novel_miR_82

Score total	:	1459
Score for star read(s)	:	3.9
Score for read counts	:	1452.6
Score for mfe	:	0.9
Score for randfold	:	1.6
Score for cons. seed	:	
Total read count	:	2861
Mature read count	:	2054
Loop read count	:	5
Star read count	:	802

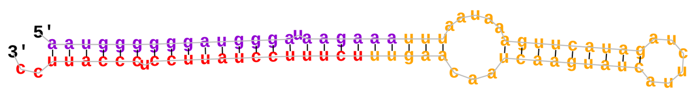

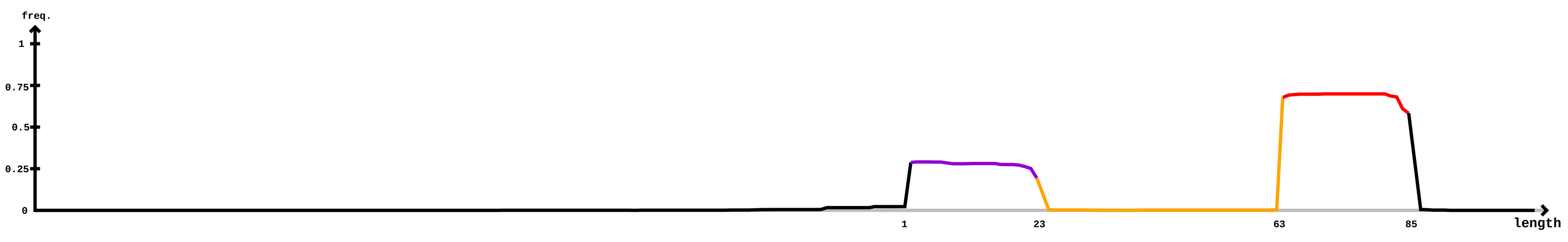

Star Mature

```
5'- agacaacuuuuuauauggucggucccaaaaucuuccucuugaaaacccuagcaaagcaaauuugcaaaaacgcauaucggcagagaaaaaaaaauugaaaacagagagacgggaaagguagcaaagaguuaggagguuauuaggaauggggggaugggauaagaaauuuaauaaaguucauagaucuuacuaugaacuaacaaguuucuuuccuauuccucccauuccauucaauuuccuaauuuccuuc  -3'  obs
    agacaacuuuuuauauggucggucccaaaaucuuccucuugaaaacccuagcaaagcaaauuugcaaaaacgcauaucggcagagaaaaaaaaauugaaaacagagagacgggaaagguagcaaagaguuaggagguuauuaggaauggggggaugggauaagaaauuuaauaaaguucauagaucuuacuaugaacuaacaaguuucuuuccuauuccucccauuccauucaauuuccuaauuuccuuc       exp
    .(((.(((........)))..)))........((((((((.........(((((.....)))))......((......))......................)))).))))((((.......((((((((((((....((((((((((((((((.((((((((.....((((((((......)))))))))...))))))))))))))))).))))))))....)))))))))))))))).   reads   mm   sample
    ............................................................................ucggcagagaaaaaaaaauuga........................................................................................................................................................   2   0   LCK-3
    ...................................................................................................aacagagagacgggaaagg....................................................................................................................................   3   0   LCK-3
    ..................................................................................................................aagguagcaaagaguuaggagguuauuagg..........................................................................................................   1   0   JT-3
    ..................................................................................................................aagguagcaaagaguuaggagguuauuagg..........................................................................................................   2   0   JT-2
    ......................................................................................................................uagcaaagaguuaggagguua...............................................................................................................   1   0   LT-1
    ......................................................................................................................uagcaaagaguuaggagguuauuagg..........................................................................................................   1   0   JCK-3
    ......................................................................................................................uagcaaagaguuaggagguuauuagg..........................................................................................................   2   0   JCK-2
    ......................................................................................................................uagcaaagaguuaggagguuauuagg..........................................................................................................   2   0   LT-2
    .......................................................................................................................agcaaagaguuaggagguuauuagg..........................................................................................................   2   0   LT-3
    .......................................................................................................................agcaaagaguuaggagguuauuagg..........................................................................................................   1   0   JCK-2
    .......................................................................................................................agcaaagaguuaggagguuauuagg..........................................................................................................   1   0   LT-2
    .........................................................................................................................caaagaguuaggagguuauuaggaaug......................................................................................................   1   0   LT-3
    .................................................................................................................................uaggagguuauuaggaaug......................................................................................................   1   0   JCK-1
    .................................................................................................................................uaggagguuauuaggaauggg....................................................................................................   1   0   JCK-3
    ..................................................................................................................................aggagguuauuaggaauggg....................................................................................................   8   0   JT-2
    ..................................................................................................................................aggagguuauuaggaauggg....................................................................................................   10  0   JT-3
    ..................................................................................................................................aggagguuauuaggaaugggg...................................................................................................   1   0   LT-2
    ..................................................................................................................................aggagguuauuaggaaugggg...................................................................................................   8   0   LCK-3
    ..................................................................................................................................aggagguuauuaggaaugggg...................................................................................................   4   0   JCK-2
    ..................................................................................................................................aggagguuauuaggaaugggC...................................................................................................   1   1   JCK-2
    ..........................................................................................................................................auuaggaaugggCggauggga...........................................................................................   1   1   JCK-1
    ..........................................................................................................................................auuaggaaugggCggauggga...........................................................................................   16  1   JT-1
    ..........................................................................................................................................auuaggaauggggggaugggau..........................................................................................   1   0   LT-1
    ..........................................................................................................................................auuaggaaugggCggaugggauaagaa.....................................................................................   1   1   JT-3
    ...............................................................................................................................................gaauggggggaugggauaaga......................................................................................   1   0   LT-3
    ................................................................................................................................................aaugggCggaugggauaa........................................................................................   7   1   JT-1
    ................................................................................................................................................aaugggCggaugggauaa........................................................................................   2   1   JCK-1
    ................................................................................................................................................aauggggggaugggauaag.......................................................................................   6   0   JT-2
    ................................................................................................................................................aaugggCggaugggauaag.......................................................................................   1   1   JCK-3
    ................................................................................................................................................aauggggggaugggauaag.......................................................................................   3   0   LT-2
    ................................................................................................................................................aaugggCggaugggauaag.......................................................................................   3   1   JT-3
    ................................................................................................................................................aauggggggaugggauaag.......................................................................................   7   0   JT-3
    ................................................................................................................................................aauggggggaugggauaag.......................................................................................   1   0   JCK-2
```

Star Mature

Sequence			
agacaacuuuuuauauggucggucccaaaaucuuccucuugaaaacccuagcaaagcaaauuugcaaaaacgcauaucggcagagaaaaaaaaauugaaaacagagagacgggaaagguagcaaagaguuaggagguuauuaggaaugggggaugggauaagaaauuuaauaaaguucauagaucuuacuaugaacuaacaaguuucuuuccuauuccucccauuccauucaauuuccuaauuuccuuc			
aaugggCggaugggauaag	5	1	JT-2
aaugggCggaugggauaaga	10	1	JCK-3
aaugggggaugggauaaga	1	0	LT-1
aaugggggaugggauaaga	8	0	JT-1
aaugggggaugggauaaga	1	0	JCK-1
aaugggCggaugggauaaga	8	1	JCK-2
aaugggggaugggauaaga	3	0	LT-3
aaugggggaugggauaaga	3	0	JCK-2
aaugggggaugggauaagaa	7	0	JCK-3
aaugggCggaugggauaagaa	13	1	JT-2
aaugggggaugggauaagaa	3	0	JCK-1
aaugggggaugggauaagaa	13	0	LT-2
aaugggggaugggauaagaa	4	0	JT-2
aaugggCggaugggauaagaa	25	1	JCK-3
aaugggggaugggauaagaa	8	0	JT-3
aaugggggaugggauaagaa	5	0	LT-3
aaugggCggaugggauaagaa	16	1	JT-3
aaugggggaugggauaagaa	14	0	JCK-2
aaugggCggaugggauaagaa	36	1	JCK-2
aaugggggaugggauaagaa	2	0	LT-1
aaugggggaugggauaagaa	6	0	LCK-3
aaugggCggaugggauaagaa	16	1	JCK-1
aaugggggaugggauaagaaa	9	0	LT-3
aaugggggaugggauaagaaa	5	0	JT-2
aaugggggaugggauaagaaa	14	0	JCK-3
aaugggggaugggauaagaaa	14	0	LT-2
aaugggCggaugggauaagaaa	62	1	JT-3
aaugggggaugggauaagaaU	1	1	JCK-3
aaugggggaugggauaaAaaa	1	1	JCK-2
aaugggggaugggauaagaaa	27	0	JCK-2
aaugggCggaugggauaagaaa	83	1	JCK-3
aaugggggaugggauaagaaa	12	0	JCK-1
aaugggCggaugggauaagaaa	107	1	JCK-2
aaugggggaugggauaagaaU	3	1	LCK-3
aaugggggaugggauaagaaC	2	1	LT-3
aaugggggaugggauaagaaU	2	1	JCK-2
aaugggCggaugggauaagaaa	35	1	JT-2
aaugggggaugggauaagaaa	10	0	JT-1
aaugggCggaugggauaagaaa	66	1	JCK-1
aaugggCggaugggauaagaaa	73	1	JT-1
aaugggggaugggauaagaaU	1	1	JCK-1
aaugggggaugggauaagaaa	12	0	LT-1
aaugggggaugggauaagaaa	6	0	JT-3
aaugggCggaugggauaagaaau	3	1	JCK-1
aaugggCggaugggauaagaaau	1	1	JCK-3
aaugggggaugggauaagaaUu	1	1	LT-1
aaugggCggaugggauaagaaau	3	1	JCK-2
aaugggCggaugggauaagaaauu	2	1	JCK-3
aaugggCggaugggauaagaaauu	1	1	JCK-1
augggggaugggauaagaaa	1	0	JCK-3
augggggaugggauaagaaa	2	0	JCK-1
augggCggaugggauaagaaa	5	1	JCK-2
Cggaugggauaagaaauuuaauaa	1	1	JCK-2
Cggaugggauaagaaauuuaauaa	2	1	JCK-3
ugggauaagaaauuuaauaaag	1	0	JCK-3
ugggauaagaaCuuuaauaaag	1	1	JCK-1
ugggauaagaaauuuaauaaaguuca	2	0	LCK-3
gaucuuacuaugaacuaacaaguA	5	1	LCK-3
uucuuuccuauuccucccauuc	5	0	LT-2
uucuuuccuauuccucccauuc	1	0	JCK-3
Cucuuuccuauuccucccauucc	1	1	JCK-2
Cucuuuccuauuccucccauucc	1	1	JCK-3
ucuuuccuauuccuccca	15	0	LT-3
ucuuuccuauuccuccca	3	0	LCK-3
ucuuuccuauuccuccca	1	0	JCK-3
ucuuuccuauuccuccca	1	0	JCK-2
ucuuuccuauuccuccca	7	0	JT-3
ucuuuccuauuccucccU	2	1	LT-3
ucuuuccuauuccuccca	5	0	LT-1
ucuuuccuauuccuccca	3	0	JT-2

```
                                                                                                                                                                                 Star                                                                            Mature
agacaacuuuuuauauggucggucccaaaaucuuccucuugaaaacccuagcaaagcaaauuugcaaaaacgcauaucggcagagaaaaaaaaauugaaaacagagagacgggaaagguagcaaagaguuaggagguuauuaggaaugggggaugggauaagaaauuuaauaaaguucauagaucuuacuaugaacuaacaaguuucuuuccuauuccucccauuccauucaauuuccuaauuuccuuc
.............................................................................................................................................................................................................ucuuuccuauuccucccau.........................   7     0   LCK-3
.............................................................................................................................................................................................................ucuuuccuauuccucccUu........................   1     1   LT-1
.............................................................................................................................................................................................................ucuuuccuauuccucccau.........................   2     0   LT-1
.............................................................................................................................................................................................................ucuuuccuauuccucccau.........................   4     0   LT-3
.............................................................................................................................................................................................................ucuuuccuauuccucccau.........................   3     0   LT-2
.............................................................................................................................................................................................................ucNuuccuauuccucccauu........................   1     1   LT-2
.............................................................................................................................................................................................................ucuuuccuCuuccucccauu........................   1     1   LT-3
.............................................................................................................................................................................................................ucuuuccuauuccucccauu........................   41    0   LCK-3
.............................................................................................................................................................................................................ucuuuccuauuccucccauu........................   16    0   JCK-1
.............................................................................................................................................................................................................ucuuuccuauuccucccCuu........................   1     1   LT-2
.............................................................................................................................................................................................................ucuGuccuauuccucccauu........................   1     1   JCK-1
.............................................................................................................................................................................................................ucuuuccuauuccucccauu........................   20    0   LT-1
.............................................................................................................................................................................................................ucuuuccuauuccucccauG........................   1     1   JCK-1
.............................................................................................................................................................................................................ucuuuccuauuccucccauu........................   25    0   JCK-3
.............................................................................................................................................................................................................ucuuuccuauuccucccauu........................   29    0   JCK-2
.............................................................................................................................................................................................................ucuuuccuauuccucccauu........................   31    0   LT-2
.............................................................................................................................................................................................................ucuuuccuauuccucccauu........................   27    0   LT-3
.............................................................................................................................................................................................................ucuNuccuauuccucccauu........................   1     1   JCK-1
.............................................................................................................................................................................................................ucuuuccuauuccucccauuc.......................   1     0   JT-3
.............................................................................................................................................................................................................ucuuuccuauuccucccauuA.......................   1     1   JCK-1
.............................................................................................................................................................................................................ucuuuccuauuccucccauuc.......................   8     0   LT-3
.............................................................................................................................................................................................................ucuuuccuauuccucccUuuc.......................   1     1   LT-1
.............................................................................................................................................................................................................ucuuuccuauuccucccauuc.......................   12    0   JCK-1
.............................................................................................................................................................................................................ucuuuccuauuccucccauuc.......................   1     0   JT-2
.............................................................................................................................................................................................................ucuuuccuauuccucccauuc.......................   6     0   LT-1
.............................................................................................................................................................................................................ucuuuccuauuccucccauuc.......................   12    0   LT-2
.............................................................................................................................................................................................................ucuuuccuauuccucccCuuc.......................   1     1   JCK-2
.............................................................................................................................................................................................................ucuuuccuauuccucccauuc.......................   14    0   JCK-2
.............................................................................................................................................................................................................ucuuuccuauuccucccauuc.......................   11    0   JCK-3
.............................................................................................................................................................................................................ucuuuccuauuccucccauucc......................   64    0   LCK-3
.............................................................................................................................................................................................................ucuuuccuauuccucccauucc......................   527   0   JCK-2
.............................................................................................................................................................................................................uUuuuccuauuccucccauucc......................   2     1   JCK-1
.............................................................................................................................................................................................................ucuuuccCauuccucccauucc......................   1     1   JT-1
.............................................................................................................................................................................................................ucuuuccuauucAucccauucc......................   1     1   JCK-3
.............................................................................................................................................................................................................ucuuuccuCuuccucccauucc......................   3     1   JCK-3
.............................................................................................................................................................................................................ucuuuccuauuccucccauuNc......................   2     1   JCK-1
.............................................................................................................................................................................................................uUuuuccuauuccucccauucc......................   2     1   JCK-3
.............................................................................................................................................................................................................ucuuuccCauuccucccauucc......................   1     1   JCK-2
.............................................................................................................................................................................................................ucuuucUuauuccucccauucc......................   1     1   JCK-2
.............................................................................................................................................................................................................ucuuuccuauuccucccCuucc......................   1     1   LT-2
.............................................................................................................................................................................................................ucuuuccuauuccucccauucc......................   388   0   JCK-3
.............................................................................................................................................................................................................ucuCuccuauuccucccauucc......................   1     1   JCK-2
.............................................................................................................................................................................................................ucuuuccuauuccucccCuucc......................   2     1   JCK-2
.............................................................................................................................................................................................................ucuuuccuauuccucccCuucc......................   1     1   JCK-1
.............................................................................................................................................................................................................ucuuuccuauuccucccauucc......................   22    0   JT-1
.............................................................................................................................................................................................................ucuuuccuauuccucccauucc......................   87    0   LT-2
.............................................................................................................................................................................................................uNuuuccuauuccucccauucc......................   1     1   JCK-2
.............................................................................................................................................................................................................ucuuuccuauuccucccauuUc......................   1     1   JCK-1
.............................................................................................................................................................................................................ucuuuccuauuccucccauucc......................   3     0   JT-2
.............................................................................................................................................................................................................ucuuuccuauuAcucccauucc......................   1     1   LT-3
.............................................................................................................................................................................................................ucuuuccuauuccucccCuucc......................   1     1   LCK-3
.............................................................................................................................................................................................................ucuuuccuauuccucccauucU......................   1     1   JCK-3
.............................................................................................................................................................................................................ucuuuccuCuuccucccauucc......................   1     1   JCK-1
.............................................................................................................................................................................................................ucuuuccuauuUcucccauucc......................   1     1   JCK-3
.............................................................................................................................................................................................................ucuuuccuauuccucccauucc......................   123   0   LT-1
.............................................................................................................................................................................................................ucuuuccuauucAucccauucc......................   1     1   LT-3
.............................................................................................................................................................................................................ucuuuccuauuccucccauucc......................   250   0   JCK-1
.............................................................................................................................................................................................................uUuuuccuauuccucccauucc......................   1     1   JCK-2
.............................................................................................................................................................................................................ucuuuccuauuccucccauucc......................   13    0   JT-3
.............................................................................................................................................................................................................ucuuuccuCuuccucccauucc......................   1     1   LT-1
.............................................................................................................................................................................................................ucuuuccuauucUucccauucc......................   2     1   JCK-2
.............................................................................................................................................................................................................ucuuuccuauuccucccauucc......................   140   0   LT-3
.............................................................................................................................................................................................................ucuuuccuauuccucccauuccC.....................   4     1   LT-2
.............................................................................................................................................................................................................ucuuuccuauuccucccCuucca.....................   1     1   JT-1
.............................................................................................................................................................................................................ucuuuccuauuccucccauucUa.....................   1     1   LT-3
.............................................................................................................................................................................................................ucuuuccuauuccucccauucca.....................   2     0   LT-1
.............................................................................................................................................................................................................ucuuuccuauuccucccauucca.....................   2     0   LT-3
.............................................................................................................................................................................................................ucuuuccuauuccucccauucca.....................   11    0   JT-1
.............................................................................................................................................................................................................ucuuuccuauuccucccauuccau....................   1     0   JCK-1
..............................................................................................................................................................................................................cuuuccuauuccucccauu........................   1     0   JCK-3
```

Star Mature

```
agacaacuuuuuauauggucggucccaaaaucuuccucuugaaaacccuagcaaagcaaauuugcaaaaacgcauaucggcagagaaaaaaaaauugaaaacagagagacgggaaagguagcaaagaguuaggagguuauuaggaaugggggaugggauaagaaauuuaauaaaguucauagaucuuacuaugaacuaacaaguuucuuuccuauuccucccauuccauucaauuuccuaauuuccuuc
..............................................................................................................................................................................................................cuuuccuauuccucccauu........................   11   0   LCK-3
..............................................................................................................................................................................................................cuuuccuauuccucccauuc.......................   1   0   LT-3
..............................................................................................................................................................................................................cuuuccuauuccucccauuU.......................   3   1   LT-2
..............................................................................................................................................................................................................cuuuccuauuccucccauucc......................   3   0   JCK-3
..............................................................................................................................................................................................................cuuuccuauuccucccauucc......................   4   0   JCK-1
..............................................................................................................................................................................................................cuuuccuauuccucccauucc......................   2   0   LT-1
..............................................................................................................................................................................................................cuuuccuauuccucccauucc......................   5   0   LT-3
..............................................................................................................................................................................................................cuuuccuauuccucccauucc......................   10   0   JCK-2
..............................................................................................................................................................................................................cuuuccuauuccucccauuccauu...................   4   0   LT-2
...............................................................................................................................................................................................................uuuccuauuccucccauu........................   2   0   JCK-3
...............................................................................................................................................................................................................uuuccuauuccucccauu........................   1   0   JCK-1
...............................................................................................................................................................................................................uuuccuCuuccucccauucc......................   1   1   LT-3
...............................................................................................................................................................................................................uuuccuauuccucccauucc......................   1   0   LT-1
...............................................................................................................................................................................................................uuuccuauuccucccauucc......................   1   0   LT-3
...............................................................................................................................................................................................................uuuccuauuccucccauucc......................   1   0   JCK-3
...............................................................................................................................................................................................................uuuccuauuccucccauucc......................   3   0   JCK-1
................................................................................................................................................................................................................uuccuauuccucccauucc......................   1   0   LT-3
................................................................................................................................................................................................................uuccuauuccucccauuccaC....................   2   1   JCK-2
................................................................................................................................................................................................................uuccuauuccucccauuccauu...................   1   0   JCK-3
................................................................................................................................................................................................................uuccuauuccucccauuccauu...................   2   0   JCK-1
....................................................................................................................................................................................................................uauuccucccauuccauucaa................   2   0   JCK-1
....................................................................................................................................................................................................................uauuccucccauuccauucaa................   1   0   JCK-3
....................................................................................................................................................................................................................uauuccucccauuccauucaa................   2   0   JCK-2
```
